# Enzyme-linked DNA dendrimer nanosensors for acetylcholine

**DOI:** 10.1038/srep14832

**Published:** 2015-10-07

**Authors:** Ryan Walsh, Jennifer M. Morales, Christopher G. Skipwith, Timothy T. Ruckh, Heather A. Clark

**Affiliations:** 1 Dept of Pharmaceutical Sciences, Northeastern University, 206 The Fenway, 360 Huntington Avenue, Boston, MA 02115, USA

## Abstract

It is currently difficult to measure small dynamics of molecules in the brain with high spatial and temporal resolution while connecting them to the bigger picture of brain function. A step towards understanding the underlying neural networks of the brain is the ability to sense discrete changes of acetylcholine within a synapse. Here we show an efficient method for generating acetylcholine-detecting nanosensors based on DNA dendrimer scaffolds that incorporate butyrylcholinesterase and fluorescein in a nanoscale arrangement. These nanosensors are selective for acetylcholine and reversibly respond to levels of acetylcholine in the neurophysiological range. This DNA dendrimer architecture has the potential to overcome current obstacles to sensing in the synaptic environment, including the nanoscale size constraints of the synapse and the ability to quantify the spatio-temporal fluctuations of neurotransmitter release. By combining the control of nanosensor architecture with the strategic placement of fluorescent reporters and enzymes, this novel nanosensor platform can facilitate the development of new selective imaging tools for neuroscience.

Understanding the complexity of discrete neural interactions in brain function is a current challenge of neuroscience. Neural networks consist of synaptic connections of interlaced neurons which form neural pathways^1^. Excitatory and inhibitory signals from chemical messengers^2^ modulate these pathways through acetylcholine, amino acid, and catecholamine neurotransmitters^3^. Understanding how these small changes assemble in a broad, cohesive manner adds to our current understanding of the functional architecture of brain connectivity^4^. Modern technologies such as positron emission tomography (PET) and functional magnetic resonance imaging (fMRI) have laid a foundation to understand the connectivity of neural pathways on a large scale. However, their spatio-temporal resolution is better suited to resolve neural circuit activity rather than synaptic activity^5^. The primary means of communication between neurons is comprised of synaptic neurotransmitter release followed by clearance through reuptake, diffusion and enzymatic degradation. Among neurotransmitters, acetylcholine has broadly contextualized effects because it is involved in cognition, behavior and neuromuscular control. While these processes are believed to be mainly synaptically regulated, phasic extrasynaptic stimulation may also play a role^6^; this is currently under investigation. Nanosensors capable of visualizing specific neurotransmitters within and around a synapse can add insight to synaptic signaling while adhering to the space requirements of the brain. These tools have the potential to greatly elucidate the contribution of synaptic and extrasynaptic signaling in cholinergic networks and add insight to disorders associated with cholinergic dysregulation such as Alzheimer’s disease and Lewy body disease^7^.

Current techniques for measuring neurotransmitter dynamics are subject to spatial and temporal limitations^8^ that hinder direct acquisition of information pertaining to synaptic neurotransmitter release and clearance. For example, techniques using electrodes and microelectrode arrays have excellent temporal resolution, however they are unable to access a single synapse due to the size of the probe^8^,^9^,^10^. The size of synaptic clefts differ per organism but are relatively small (300 nm × 300 nm × 20 nm)^11^,^12^ in comparison to current technologies for single neuron readings such as microelectrodes (2–5 μm)^13^. These limitations have spurred the development of new techniques for visualizing neurotransmitter dynamics that employ fluorescent tools such as small molecule indicators and genetically-encoded reporters^14^,^15^. Nevertheless the majority of these techniques are not specific for recognizing neurotransmitters and detecting changes in their concentrations^16^,^17^. Neural environments contain multiple analytes and neurotransmitters from adjacent neurons and glial cells such as acetylcholine, glutamate and dopamine^18^. Additionally, *in vivo* capillary microdialysis studies identified 29 additional neuropeptide and protein neuromodulators present in brain extracellular fluid^19^. This requires sensing technologies to be selective for a molecule of interest without interference from other molecules present in the fluid.

Enzyme-based sensors are widely used to elucidate biological processes and networks with predictable substrate selectivity. This enables the design of a sensor specific for acetylcholine that is easily introduced into biological systems. Many advances in enzyme-based sensors are macrosensors, electrodes or arrays that are not suitable for measuring discrete changes occurring in confined spaces. This field can be enhanced by implementation of nano-sized enzyme sensors tailored for constrained environments such as the synaptic cleft^5^. Single molecule fluorescent indicators analogous to fluo- and indo- dyes for Ca^2+^ do not exist for acetylcholine because a selective small molecule recognition element for acetylcholine is not available. However, enzymes such as butyrylcholinesterase selectively bind to and hydrolyze acetylcholine. Enzymatic hydrolysis locally reduces pH, making it possible to detect acetylcholine by pairing the enzyme with a pH-sensitive fluorescent indicator^20^.

DNA nanotechnology has proven particularly useful for applications requiring functional nanostructures and complex nano-scale arrangements, such as DNA origami^21^ and DNA walkers^22^. The use of DNA in our platform promotes biocompatibility, ease of design, self-assembly, high surface area to volume ratio and tethers molecules in defined arrangements^23^. Double-stranded DNA dendrimer scaffolds are grown from an initial seed structure through the assembly of DNA layers. The dendrimer size is tailored by adding successive DNA layers with complementary sticky ends or terminating the structure. The resulting DNA dendrimer scaffolds have a considerably larger surface area to volume ratio than micelles or liposomes of similar diameters. This enables the strategic placement of sensing components and fluorescent probes within the dendrimers.

DNA-based sensors have many advantages such as high solubility in water, commercial availability and ease of synthesis. Additionally, naked DNA has very low cell membrane permeability due to its negative charge^24^,^25^. As an extracellular nanosensor platform, this impermeability translates to prevent endocytosis. The majority of DNA-based sensors are designed with DNA-modifying enzymes that use the DNA backbone as a substrate. These types of sensors utilize fluorophores conjugated to nucleotides and have been found to be highly robust in studies with DNA sequencing and protein analysis^20^.

Here, we developed DNA dendrimers containing evenly spaced, covalently linked fluorescein molecules and enzymes to produce nanosensors (Fig. 1)^26^. This structural arrangement mitigates fluorophore self-quenching^27^,^28^ and tunes the sensitivity and dynamic range to the physiological concentrations of acetylcholine. The combined spatial control and simple assembly make the DNA dendrimers an excellent platform for developing tailored nanosensors based on enzymatic small molecule recognition. Our DNA dendrimer nanosensor is designed to fit in confined extracellular spaces and can easily be modified for use with any enzyme system.

## Results and Discussion

**Figure 1:**
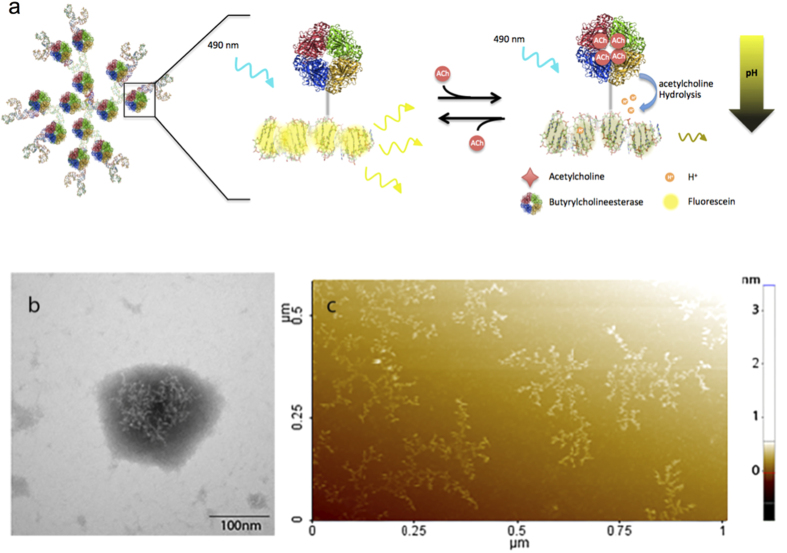
(**a**) A computationally rendered representation of the nanosensor and schematic of the proposed mechanism of DNA dendrimer-based nanosensors. The nanosensor design consists of 81 fluorescein molecules tethered in close proximity to 12 butyrylcholinesterase molecules using a DNA dendrimer calculated to be approximately 100 nm in diameter. The DNA nanosensor is highly fluorescent in the absence of acetylcholine when excited at 490 nm. In the presence of acetylcholine, the butyrylcholinesterase hydrolyzes acetylcholine, reducing the local pH and causing a decrease in the fluorescence of fluorescein. Structural characterization of the DNA dendrimer was performed using (**b**) TEM images of the structure, indicating diameters of 130 ± 24 nm, and with (**c**) AFM analysis of pre-assembled DNA dendrimer structures deposited on a mica surface.

To utilize this platform to produce functional acetylcholine nanosensors, we evenly spaced butyrylcholinesterase and fluorescein on the DNA dendrimer subunits. Fluorescein is easy to attach to DNA and is widely used in biological applications due to its sensitive pH-dependent fluorescence and pK_a_ of 6.4. Although fluorescein is prone to photobleaching, its high quantum efficiency and six-fold increase in fluorescence between pH 6–8^29^,^30^ make it an excellent fit for this nanosensor design. These nanosensors self-assemble through base pairing into a predicted branched structure with binding sites for 81 evenly spaced fluorescein molecules and 12 cholinesterase enzymes (Fig. 1a), and were responsive to acetylcholine at low millimolar concentrations (Supplementary Figure S1).

Although acetylcholinesterase has a higher affinity for acetylcholine in comparison to butyrylcholinesterase (Supplementary Figure S2), it showed a well-known substrate inhibition effect that resulted in a decrease in response at concentrations beyond the low millimolar range. This demonstrates that butyrylcholinesterase-based sensors were more responsive to millimolar concentrations of acetylcholine in the upper physiological range. The novelty of the sensor’s modular design is in the ability to easily exchange the recognition enzyme to acetylcholinesterase in later studies. This adjustment could produce sensors with a linear range at lower acetylcholine concentrations, if desired.

Transmission electron microscopy (TEM) was used to confirm the size of the nanosensors. Nanosensor diameters were 130 ± 24 nm (Fig. 1b), consistent with the calculated 100 nm diameter of the DNA dendrimers. The large spread in the DNA dendrimer size can be attributed to the flexibility of the DNA. Although TEM images with negative stain are not optimal because the stain obscures structural detail due to shadowing, positive staining procedures are not reliable for DNA. To visualize the branching structure, acetylcholine nanosensors were imaged with non-contact mode Atomic Force Microscopy (nc-AFM), or frequency modulation detection. Images of the nanosensors clearly show the highly branched structures of the DNA dendrimers (Fig. 1c) including some of the smaller DNA constituents used for self-assembly into dendrimer structures. Nc-AFM was used to resolve the structure of dried DNA dendrimer samples on (3-Aminopropyl) triethoxysilane-functionalized mica to decrease deformation. However, since the DNA was dried onto a functionalized mica substrate, distortions may have occurred resulting from dendrimer aggregation and layering^31^. Thus TEM images more accurately represent the size of the nanosensors and nc-AFM discerns the branching structure of the DNA dendrimers^32^. Nc-AFM and TEM images show that DNA functionalized with butyrylcholinesterase and fluorescein self-assemble into the predicted designed DNA dendrimer structures.

**Figure 2:**
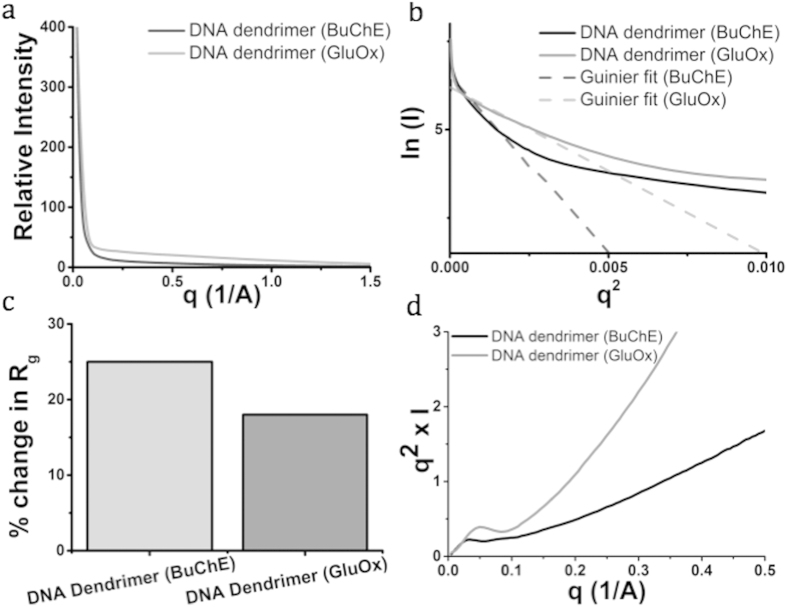
Small angle x-ray scattering (SAXS) of DNA dendrimers with butyrylcholinesterase (BuChE) or glucose oxidase (GluOx) attached was done to further assess the structure and stability of the DNA dendrimers. We performed SAXS with two samples of DNA dendrimers (at 1 mg/mL in PBS). (**a**) SAXS scattering data depicting the standard plots of circularly averaged intensity as a function of *q*, where *q* = 4π/λ[sin(θ/2)] (θ = scattering angle). (**b**) Guinier plot of scattering data demonstrates the conformational flexibility of the dendrimer structures and yields an approximate size based on the Guinier slopes. (**c**) Quantified percent change in the radius of gyration (R_g_), measured by the change in the R_g_ of the experimentally-determined value from the theoretical value. (**d**) Kratky plot of scattering data indicates the heterogeneous internal substructure of the DNA dendrimers.

**Figure 3:**
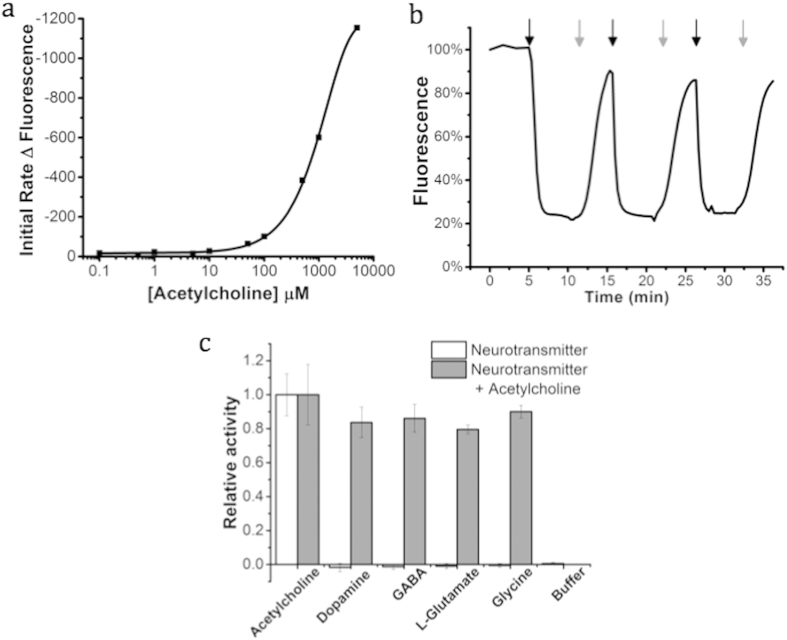
To assess the responsiveness of the sensors to acetylcholine, (**a**) initial rates over 15 seconds produced by 5.6 nM solutions of the sensors in phosphate buffer saline were determined (K_m_ = 1.05 mM, *n* = 3). (**b**) The florescent signal of the nanosensors (380 nM) was monitored in response to repeated cycles of exposure to 50 mM acetylcholine in PBS (Black arrow) followed by PBS washes (Grey arrow) demonstrating their reversible response to acutely high concentrations of acetylcholine. The (**c**) selectivity of the nanosensors was examined by exposing them to neurotransmitter solutions (30 mM) (White bars) of GABA, L-glutamate, glycine, and dopamine (300 μM) (ANOVA, *p < 0.005 for *n* = 3). The activity of the sensors towards acetylcholine in the presence of the neurotransmitters was then examined with a successive addition of 15 mM acetylcholine (Grey bars) (ANOVA, *p > 0.05 for *n* = 3). The acetylcholine response range for butyrylcholinesterase based sensors includes the expected millimolar synaptic acetylcholine concentrations^9^, and the sensors responses may be further enhanced by the presence of endogenous synaptic cholinesterases^51^. All data are shown as mean ± s.d.

Solution small angle x-ray scattering (SAXS) provides direct structural information about the DNA dendrimer structure. It has been shown that well-ordered proteins and packaged DNA show highly-resolved scattering profiles^33^. We collected SAXS scattering data for DNA dendrimers with butyrylcholinesterase attached (DNA-BuChE) and DNA dendrimers with the more stable glucose oxidase (DNA-GluOx) attached in phosphate buffered saline pH 7.4 (PBS) to investigate the internal structure of the DNA dendrimers and determine the extent of enzyme conjugation in the structures (Fig. 2). We chose glucose oxidase as a control since it is well characterized, stable in solution and simple to attach in our system, although it is smaller than butyrylcholinesterase (160 kDa versus 440 kDa for BuChE). Figure 3a shows the standard plots of circularly averaged intensity as a function of *q*, both traces display a distinct diffraction peak associated only with DNA packaging. The position of the diffraction peak is determined by the DNA spacing. Less ordered DNA structures will exhibit less coherent diffraction, leading to decreased DNA peak area. Increasing DNA flexibility leads to less bending stress, resulting in decreased repulsive interactions and enhanced mobility of the dendrimer. Indeed, our SAXS data show that the DNA-BuChE construct has a decreased peak area relative to the DNA-GluOx construct, indicating that it is less ordered, and likely more flexible (Fig. 3a). Simultaneously, the area of the DNA scattering peak decreases by ~6-fold (Fig. 2a).

The Guinier approximation estimates the shape and size of objects by analysis of SAXS scattering at small angles. The size of the resolved structures provides details about the flexible examined regions shown in the Guinier plots of the SAXS data (Fig. 2b). The resolved area of the Guinier plots for the DNA dendrimers is curved at low scattering angles, consistent with either aggregation or a flexible structure (Fig. 2b). Fitting the DNA-BuChE plot to a two-parameter regression suggests a radius of gyration (R_g_) of 55 Å, corresponding to a solid sphere of radius 71 Å (142 Å diameter) (Fig. 2c). For DNA-GluOx, the Guinier plot is more distinguished and correlates to R_g_ of 37 Å, corresponding to a solid sphere radius of 48 Å (100 Å diameter) (Fig. 2c). These sizes are consistent with a single arm extending from the initial layer of three-way junctions, containing one three-way junction and one conjugated enzyme. Thus there is one enzyme per dendrimer section, which is consistent with the nanosensor design. It is reasonable to infer less densely packaged DNA and weaker repulsive interactions lead to a more mobile DNA construct. This is supported by the larger increase in the R_g_ (in comparison to the theoretical R_g_ for the protein size) for the DNA-BuChE construct (Fig. 2c) due to the larger size of enzyme attached. Additionally, the Kratky plot resolves dendrimer branching density and heterogeneity of secondary structure. The Kratky plot demonstrates DNA-BuChE has no well-defined maximum (Fig. 2d), which is consistent with a heterogeneous population of particles with varying internal structure. However the size is homogeneous according to the Guinier plot (Fig. 2b). DNA-GluOx has a real maximum (Fig. 2d), which is indicative of less heterogeneous substructures than DNA-BuChE. Thus the DNA-BuChE construct may not fill all of the enzyme attachment sites, possibly due to size constraints, whereas the DNA-GluOx is a smaller enzyme that may fill all of its sites, leading to more homogenous substructures.

Acetylcholine concentration in the extracellular fluid of the brain is in the low nanomolar range^34^,^35^ and thought to peak in the low millimolar range during synaptic transmission^9^. Computational studies have concluded synaptic concentrations of free acetylcholine below 0.9 mM to likely be inadequate for postsynaptic receptor activation^36^. Our goal was to produce a fluorescent indicator of transiently elevated acetylcholine levels; so ideally, the dynamic range of our sensors would be linear at low millimolar concentrations. To characterize the concentration-dependent response of the nanosensors to acetylcholine, the nanosensors were calibrated in solution from 0.1 μM–5 mM acetylcholine. Acetylcholine recognition and hydrolysis by butyrylcholinesterase conjugated to the DNA dendrimers decreases the local pH, causing a concentration-dependent rate of change in fluorescence intensity (Fig. 3a). In this assay, the initial rate of fluorescence change was monitored during a 30 second incubation of nanosensors and acetylcholine. The rate of fluorescence change increases with substrate concentration, as expected, and the sensors respond dynamically to acetylcholine with a 200 μM lower limit of detection (Fig. 3a).

For any practical application, sensor reversibility is necessary to continuously visualize acetylcholine dynamics. To assess reversibility, we sealed the nanosensors inside dialysis tubing, secured the assembly to glass coverslips, and imaged them with confocal microscopy. Cyclic perfusion between 50 mM and 0 mM acetylcholine leads to a decrease in fluorescence followed by recovery of fluorescence (Fig. 3b). This demonstrates that the activity of the nanosensors is conserved despite pH decreases resulting from acetylcholine hydrolysis, confirming local pH changes do not cause irreversible nanosensor degradation (Fig. 3b). The observed response time is not a measure of the response time of the nanosensors, but rather the diffusion time of solution into the dialysis tube. To ensure the nanosensors respond to localized acetylcholine rather than bulk pH, solutions were independently monitored for pH changes (Supplementary Figure S8). Although 50 mM supersedes physiological levels of acetylcholine, it was chosen to maximize the pH change for assessing the potential for degradation due to acidic conditions.

Nanosensor stability was determined by measuring the responsiveness of the nanosensors to acetylcholine after 2 months of storage at 4 °C. Nanosensors retain 84% of their initial activity (Supplementary Figure S3) showing that the DNA dendrimer construct is a stable arrangement for all components. To determine the photostability of fluorescein incorporated into the structures, the DNA dendrimers with fluorescein were embedded in agar and photobleached with continuous laser excitation. As expected, fluorescein in the DNA dendrimers and free fluorescein are equally susceptible to photobleaching (Supplementary Figure S4). The quantum yield of the fluorescein-labeled nanostructure (0.71) is consistent with the quantum yield of fluorescein (0.79) and maintains the fluorescence change per pH unit (Supplementary Figure S5, S6). As we expected, covalently attaching fluorescein to the DNA scaffold did not negatively affect the properties of the dye, but it is clear that dyes with better photobleaching properties would improve the nanosensors for cellular imaging in the future.

Selectivity is paramount for the nanosensors to produce reliable data in biological media, and enzymatic recognition elements are attractive due to their inherently high selectivity. To demonstrate high nanosensor selectively, we measured the response to common amino acid and catecholamine neurotransmitters^37^, as well as the response to acetylcholine in the presence of the neurotransmitters (Fig. 3c). We found the nanosensors did not respond to amino acid neurotransmitters tested, including gamma-aminobutyric acid (GABA), L-glutamate and glycine (p < 0.005). In addition, the presence of GABA, L-gluatamate and glycine had no effect on nanosensor responses to acetylcholine, which demonstrated that they did not inhibit the enzymatic response to acetylcholine (Fig. 3c). Initially dopamine (catecholamine) did interfere with the response of the nanosensors to acetylcholine. Millimolar concentrations of dopamine significantly decreased nanosensor responsiveness through nonspecific quenching of fluorescein (Supplementary Figure S7). Though millimolar dopamine concentrations fall outside of the expected physiological range, re-examination of the nanosensors in presence of predicted upper physiological dopamine levels (300µM) showed no interference (Fig. 3c)^38^.

**Figure 4:**
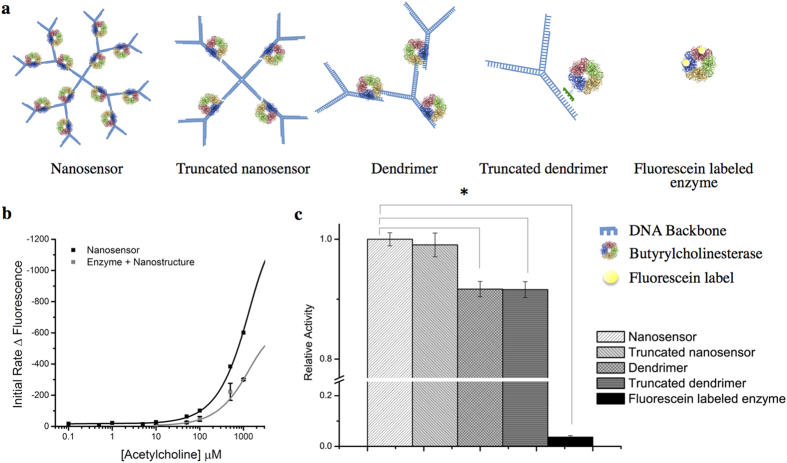
(**a**) Largest (left) to smallest (right) schematics of the nanosensor structures with associated enzymes. (**b**) Initial rates of nanosensors and butyrylcholinesterase in solution with fluorescein labeled DNA dendrimers were determined in response to acetylcholine in the physiological range (K_m_ = 1.05 mM, *n* = 3, black line; K_m_ = 1.05 mM, *n* = 3, gray line). (**c**) The relative activity of nanosensor subunits and truncated nanosensors in the presence of 1 mM acetylcholine was tested. Single dendrimers, truncated dendrimers and fluorescein labeled enzyme showed a significant decrease in activity (*p* < 0.005, *n* = 3) in comparison to full nanosensors. Initial rates calculated at 30 seconds for all trials.

Careful spacing of the enzyme and fluorophores on the DNA dendrimer structure subunits is critical to the response characteristics of the nanosensor. We constructed several variants of our design to demonstrate that the DNA scaffold is essential for maximizing the nanosensor response (Fig. 4a). First, DNA scaffolds linked to fluorescein were simply mixed with untethered butyrylcholinesterase in solution and calibrated for their response to acetylcholine. Fully assembled nanosensors display a two-fold increase in sensitivity in comparison to equivalent concentrations of free butyrylcholinesterase in solution with fluorescein-labeled dendrimers (Fig. 4b). This change in sensitivity is evident by the steeper slope of the calibration curve, showing the individual components of the sensor are not as effective as the fully assembled architecture. Second, we directly linked fluorescein to butyrylcholinesterase with no DNA scaffold. Butyrylcholinesterase was functionalized with up to seven fluorescein molecules; the most dynamic contained two conjugated fluorescein molecules, which minimized quenching. Nanosensors built with the DNA scaffold displayed a 27-fold increase in activity in the presence of acetylcholine when compared to an equivalent amount of fluorescein-bound enzyme.

To determine the smallest nanosensor subunit required to maintain response, all subunit arrangements shown in Fig. 4a were tested for activity in contrast to the full 100 nm nanosensors and fluorescein-labeled enzyme alone. Truncated nanosensors did not show a significant loss of activity in comparison to full nanosensors (Fig. 4c). In addition, this structure is 32 nm in diameter, which could be useful for the constrained spaces that would be encountered in extracellular environments. Conversely, nanosensor subunits containing less than four enzymes showed a significant decrease in activity: single dendrimers, truncated dendrimers and fluorescein labeled enzyme (*p* < 0.005, *n* = 3). These results clearly demonstrate that arranging enzymes and fluorescent reporters in close proximity create a localized concentration effect and a means for tuning nanosensor sensitivity in future studies.

**Figure 5:**
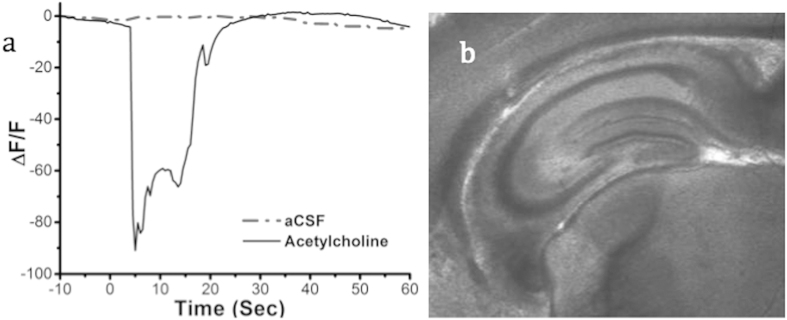
(**a**) Nanosensor responsiveness and reversibility in an acute mouse cortex brain slice in response to acetylcholine perfusion. All data are shown as mean ± s.d. (**b**) Image of acute mouse brain slice used for imaging.

One of the potential drawbacks of the dendrimer structure is that the enzymes and dye molecules are not well shielded from potential fouling and degradation in a cellular environment. To demonstrate that nanosensors remain active in a cellular environment, nanosensors were injected 50 μm deep into layer 4 of the cortex in tissue slices. After 5 minutes to allow for potential diffusion away from the injection site, a solution of 50 mM acetylcholine was perfused into the imaging chamber for 15 seconds. Nanosensor fluorescence decreased by 90% in response to perfused acetylcholine (Fig. 5). Nanosensor fluorescence recovered when acetylcholine-free aCSF was then perfused over the tissue slice. The reversibility measurement in tissue was important because the nanosensors fluorescence decreases when the analyte is increased, also called a “turn-off” sensor. In these tissue studies, reversibility demonstrates the decrease in signal was not due to degradation of the sensor.

## Conclusion

Our nanosensors overcome current obstacles to sensing acetylcholine in the neural environment by having a fast reaction time and nano-scale size. We show the nanosensor responses to acetylcholine with high sensitivity, selectivity, and reversibility in the neurophysiological range. This demonstrates that modular DNA dendrimers are a powerful platform for designing nanosensors. DNA nanosensors self assemble with intact biological components and capitalize on the exquisite specificity of biological recognition elements. Though electrode sensors for acetylcholine have used this approach with the muscarinic receptor^39^, our nanosensors combine biological specificity with the size and responsiveness of nanoparticle-based nanosensors^40^,^41^. Although the nanosensor platform is designed for use in the synaptic environment the DNA dendrimers can be adapted for other purposes. The platform’s modular nature makes it adaptable to other small molecules and bio-analytes that are difficult to measure in biological systems. In the future, targeting nanosensors to cholinergic synapses would be valuable for further studies. For instance, conjugation of nicotinic receptor antagonists, such as α-Bungarotoxin, to the DNA backbone would target the nanosensors to alpha7 nicotinic acetylcholine receptors on the synaptic cleft of cholinergic post-synaptic neurons. Additionally, this platform can be modified with additional fluorophores and enzymes to sense more than one neurotransmitter or analyte for future tissue studies with 2-photon microscopy to monitor changes in neuron activity.

## Materials and Methods

*Equine* butyrylcholinesterase (EC 3.1.1.8) was acquired through the generous donation of Dr. Oksana Lockridge or alternatively purchased from Sigma. Acetylcholinesterase from *Electrophorus electricus* (EC 3.1.1.7), glucose oxidase from *Aspergillus niger* (EC 1.1.3.4) and all other reagents were acquired from Sigma unless otherwise noted. All DNA sequences were purchased from Alpha DNA.

### DNA dendrimer design

The DNA dendrimer scaffold for the enzyme based nanosensor was designed to enable efficient DNA annealing for a branched structure. Preliminary sequences were obtained by using a random sequence generator^42^, designed in a three part layering system and tailored to assemble with predictable secondary structure as confirmed by RNAfold^43^ minimum free energy calculations. DNA junctions and complementary sticky ends were produced with the aid of a reverse complement generator^42^ and jointly submitted to RNAfold^43^ to confirm the secondary structure produced by base pairing. The sequences include Fluorescein-dT tags equally spaced along the backbone of the structure. Additional C8-Alkyne-dT modifications, for click tethering of the enzyme, were added to short complementary DNA sequences to enable site-specific incorporation of the cholinesterases along the backbone of the structure. The rendered DNA nanostructure was prepared using Pymol^44^, and the deposited crystal structure of human butyrylcholinesterase (PDB ID: 1P0P)^45^,^46^.

### DNA dendrimer nanosensor assembly

DNA nanostructures were assembled based on the procedures described by Zhou *et al.*^23^. All of the DNA sequences were dissolved in 0.1 M phosphate buffer, pH 7.4, to a concentration of 100 μM, heated to 90 °C for five minutes and allowed to cool before use. Initially the four sequences for the central four-way junction were mixed in equal molar amounts. Separately, the sequences for the three-way junction which comprises the first growth ring of the DNA dendrimer and the enzyme-tagged sequence were mixed in equal molar amounts to create a pool with four times the number of moles produced for the seed four-way junction. Sequences for the three-way junction comprising the second growth ring of the DNA dendrimer and the enzyme-tagged sequence were combined in the same way but at eight-fold the number of moles used to produce the seed four-way junction. The components for the first growth ring and the four-way junction were combined and the structures were incubated for two hours at room temperature to enable assembly. Components for the second growth ring were added and the structure was allowed to assemble for two hours at room temperature before use or storage at 4 °C.

### Preparation of cholinesterases for click chemistry

A solution of 0.1 M 15-Azido-4,7,10,13-tetraoxapentadecanoic acid (Alfa Aesar) in dimethylformamide (DMF) (1 μL) was added to 24 μL DMF containing 20 μM *N*, *N*, *N*′, *N*′-tetramethyl-*O*-(*N*-succinimidyl)uranium tetrafluoroborate (Sigma) and 40 μM 4-Dimethylaminopyridine (Sigma). The solution was incubated on a shaker for 60 minutes at room temperature. Following the incubation the solution was added to 975 μL 0.1 M phosphate buffer, pH 8, containing 1 nmol of the cholinesterases and incubated for 2 hours at room temperature. The sample was then washed 3 times using microfilter centrifuge tubes, (Millipore MWCO 50 kD), by centrifuging for 10 minutes 15,000 × *g*, with 50 mM Tris-HCl, pH 8, to prepare it for click chemistry. The final volume was adjusted to 50 μL with 50 mM Tris-HCl, pH 8.

### Click chemistry

The click chemistry reaction between the azide-labeled cholinesterases and the alkyne-labeled DNA was performed using the Click-iT^®^ Protein Reaction Buffer Kit (Invitrogen) according to the manufacturer’s instructions. Briefly, the 50 μL azide-labeled enzyme sample was combined with 100 μL Click-iT^®^ reaction buffer containing 4 nmol of the alkyne labeled DNA and 10 μL of DI H_2_O. The sample was mixed prior to the addition of 10 μL of the kits CuSO_4_ solution. The reaction was initiated with the addition of 10 μL of additive 1, reacted for 3 minutes and finished with the addition of 20 μL of additive 2. The sample was washed 12 times using microfilter centrifuge tubes (Millipore MWCO 100 kD), by centrifuging for 8 minutes, 14,000 × *g* at 10 °C, with 0.1 M phosphate buffer, pH 8.

### Attachment of glucose oxidase to DNA

The attachment of glucose oxidase to DNA dendrimers was done in a two-step process. First, preparation of glucose oxidase for click chemistry was preformed as described above with cholinesterases. Second, the azide-labeled glucose oxidase are attached to dibenzylcyclooctyne-labeled DNA by copper free click chemistry. The attachment was performed by mixing 4 nmol of the dibenzylcyclooctyne-labeled DNA with azide-labeled glucose oxidase and incubating the reaction mixture at 4 °C for 24 hours.

### Quantum yield determination

Quantum yield was calculated as described in Würth *et al.*^47^. Fluorescence and absorbance data were collected on a Model 814 Photomultiplier Detection System (Photon Technology International) and Spectramax M3 Mulit-mode microplate reader (Molecular Devices), respectively. Data analysis was done using a|e—UV-Vis-IR Spectral Software version 1.2 (FluorTools) with fluorescein isothiocynate (FITC) as a standard.

### TEM structural determination

Samples were prepared using the procedure described by Bock *et al.*^48^. Briefly, the DNA nanosensors were washed with distilled water to remove buffer salts. Once the structure was sufficiently salt free a 300-mesh carbon film-coated copper grid (Electron Microscopy Sciences) was placed on a 10 μL, 0.6 pM drop of the DNA nanosensors for 2 minutes. The grid was washed three times with distilled water and placed onto a 5 μL drop of 1.5% phosphotungstic acid stain (Electron Microscopy Sciences) for an additional 3 minutes. The grid was dried with filter paper to remove excess liquid. Images were acquired in dark-field mode using a JEOL 1010 TEM at 80 kV accelerating voltage.

### AFM structural determination

Samples for study by AFM were prepared using the procedure described by Lyubchenko *et al.*^49^. Briefly, slices of freshly cleaved mica (Electron Microscopy Sciences) were placed in a desiccator in the presence of 3-aminopropyltriethoxysilane (Sigma) and *N*,*N*-Disopropylethylamine (Sigma) for 2 hours. The mica was dried under an argon atmosphere for an additional 2 hours at room temperature. Approximately 0.2 μg/mL of the DNA nanostructure was added to the APTES-functionalized mica for 3 minutes. After DNA samples were attached to the mica surface, the surface was washed with 60 μL of 2 mM magnesium acetate solution and dried with condensed air. The mica was further dried under an argon atmosphere for an additional 2 hours. Imaging was performed with non-contact mode AFM on a Park Scientific NX10 using a Z-scanner bandwidth of 5.23 Hz, with PPP-NCHR tips (Park Scientific).

### Small-Angle X-Ray Scattering

DNA dendrimers structures with butyrylcholinesterase attached (DNA-BuChE) and DNA dendrimers with the more stable glucose oxidase (DNA-GluOx) attached in PBS were probed by SAXS at room temperature. Each of the samples was made at a concentration of 1 mg/mL in PBS. SAXS measurements were performed using Beamline NSLS-X9 at the Brookhaven National Laboratory, under proposal number PASS 28232. Incident X-ray wavelength (λ) was 1.33 Å and a two-dimensional CCD detector was used to collect the scattering intensity data. The intensity profile was output as the plot of the scattering intensity (I) vs. the scattering vector, *q* = 4π/λ[sin(θ/2)] (θ = scattering angle).

### Esterase Activity

Nanosensor fluorescence was assessed using a Synergy H1 Hybrid Multi-Mode plate reader (BioTek) by exciting the structures at 485 nm and monitoring the emission at 520 nm in the presence of acetylcholine concentrations varying from 0.1 μM to 50 mM. For calibration curves, fluorescence values were taken every 1 second for 60 seconds. Calibration curves were generated by calculating the initial rates of the kinetic traces generated for each acetylcholine concentration exposure over the first 30 seconds. Nanosensor assemblies of the different dendrimer structures were analyzed by normalization of the change in rates over thirty seconds due to differing concentrations of fluorescein conjugated DNA. The change in fluorescence response was normalized for each well and the change in response to initial fluorescence rate was determined by the averaged normalized values per well. Activity rates are shown as the total change in rate of fluorescence per assay in positive values to clearly illustrate the data. All assays used a fresh solution of acetylcholine in <4 hours. The pH of acetylcholine solutions were tested over time to validate the specific response to acetylcholine, rather than bulk pH (Supplementary Figure S8). Reversibility studies were performed by placing the nanosensors in microdialysis tubing (Spectra/Por^®^*in vivo* microdialysis tubing MWCO 13 kD) and mounting the tubing on a slide. Samples were then examined using a Zeiss LSM 700 confocal laser scanning microscope, excitation 488 nm, with sequential exposures to alternating solutions of acetylcholine and buffer. Photobleaching was performed by spotting a glass slide with a sample of either the DNA dendrimer or fluorescein (20 μM), samples were dried before a drop of 1.5% agar was solidified over the samples. Samples were bleached using a 488 nm laser on a Zeiss LSM 700.

### Animal Research

All animal experiments were approved by the Northeastern University’s Institutional Animal Care and Use Committee (IACUC) and carried out in accordance with the approved guidelines.

### Brain Slice Preparation

Coronal slices were cut from the brain of CD-1 IGS mice (Charles River Laboratories International, Inc., Wilmington, MA) between 6–21 days old. Anesthetized mice (2% isoflurane in O_2_) were sacrificed by cervical dislocation and the brain was immediately removed. A coronal blocking cut was made to remove the cerebellum prior to cutting. 300 μm-thick coronal brain slices were cut using a vibratome (Campden Instruments Ltd., Loughborough, England) and then immediately transferred to the NMDG recovery solution for 15 minutes at 23 °C^50^. The slices were then transferred to artificial cerebral spinal fluid (aCSF) to recover for an additional 45 minutes at 23 °C. Throughout both incubation steps, the media were bubbled with carbogen gas (95% O_2_/5% CO_2_). After the one hour recovery, a brain slice was transferred to the imaging chamber for injection and imaging.

For nanosensor injection, a pulled glass pipette (tip opening ~800 nm) was filled with 4 μL of nanosensors in aCSF. A digital micromanipulator (Sutter Instruments) positioned the pipette tip 50 μm below the slice surface using the diagonal approach mode. Nanosensors were gently injected using a constant positive pressure for five seconds, and then the pipette tip was withdrawn while maintaining positive pressure on the pipette tip. Nanosensors’ diffusion away from the injection site was monitored by acquiring images every second until the fluorescent signal from the injection region had ceased to decrease.

Nanosensor responses to perfused acetylcholine were measured by acquiring 2 minute videos, with acquisitions at 2 Hz. The first 10 seconds of a video imaged the nanosensors under perfusion with standard aCSF followed by a 15 second rapid perfusion of 5 mL aCSF with 0 or 50 mM acetylcholine. After the rapid perfusion, standard aCSF was perfused back into the imaging chamber to clear the acetylcholine.

Image exposure times were determined prior to acquiring images to ensure that the maximum grayscale value was less than half of the 16-bit maximum. Images were analyzed by first subtracting the mean value of the background, acquired prior to nanosensor injection, from the image sets. Because nanosensors were distributed throughout the field of view, a large central ROI was selected and the average grayscale value was determined for the ROI in each frame.

### DNA dendrimer fluorescence change with pH

DNA dendrimers were assembled piece wise without tethered enzyme. Dendrimer structures were tested against a pH range of 3–9, in triplicate. Fluorescent spectrums were acquired by Spectramax Gemini EM microplate fluorometer (Molecular Devices). Dendrimers were brought up to 0.24 mM concentrations in Phosphate buffered saline pH solutions (pH 3–9). Florescent measurements were taken in a Nunc 384-Well Optical Bottom Plates (Thermo Scientific) with an excitation of 494 nm and emission recorded at 520 nm.

### Data analysis

Responsiveness of the sensors was examined over a range of acetylcholine concentrations from 0.1 μM to 5 mM, with each point repeated 3 times. Limits of detection were determined using linear regression at each end of the calibration curve. The limits were calculated by dividing the standard error of the regression by the slope and multiplied by three. Nonlinear regression of the data to the Michaelis-Menten equation produced an *r*^2^ value of 0.99, for data points obtained within the limits of detection. All calibration curve data was recorded as initial rate of enzymatic activity during the first 30 seconds after substrate addition.

ANOVA was used to examine interference produced by other neurotransmitters. To determine if the sensors were responsive to other neurotransmitters, changes in fluorescence were examined following exposed to 30 mM concentrations of various neurotransmitters, *p* < 0.005 (*n* = 3). The samples were further analyzed following the addition of acetylcholine, final concentration of 15 mM, to determine if the other neurotransmitters interfered with the responsiveness of the sensors to acetylcholine, *p* < 0.005.

## Additional Information

**How to cite this article**: Walsh, R. *et al.* Enzyme-linked DNA dendrimer nanosensors for acetylcholine. *Sci. Rep.* **5**, 14832; doi: 10.1038/srep14832 (2015).

## Supplementary Material

Supplementary Information
