# Supplementary material for: Enzyme-linked DNA dendrimer nanosensors for acetylcholine
Source: Sci Rep. 2015 Oct 7;5:14832. doi: 10.1038/srep14832 (PMC4595838; doi:10.1038/srep14832)
Supplement: Supplementary Information [file srep14832-s1.pdf]

Supporting Information

Enzyme linked DNA dendrimers as nanosensors for acetylcholine

*Ryan Walsh‡, Jennifer M. Morales‡, Christopher G. Skipwith, Timothy T. Ruckh, and Heather A. Clark**

Dept of Pharmaceutical Sciences, Northeastern University, 206 The Fenway, 360 Huntington Avenue, Boston, MA 02115, United States

‡These authors contributed equally to this work.

DNA sequences

Section 1: 4 way junction

ATAATTCAGCGATAATCAATCAAATCAGTTTCTGTTCGGCATATTCAACCAGTT
ATAATTCAGCGATAACTGGTTGAATATGCCGAATTCTGTACTATTGTAAAATAT
ATAATTCAGCGATATATTTTACAATAGTACAGAGTTAACTCATTTGCGTCTAAT
ATAATTCAGCGATATTAGACGCAAATGAGTTAAGAGAAACTGATTTGATTGATT

Features
Common sticky end
ATAATTCAGCGAT
Placement of Fluorescein labeled bases
T

Section 2: 3 way junction

ATCGCTGAATTATCACGCATCACCATCGATACAATTTTATAGAGCAGTAT
CGCAAGCACAACAAGAGAATAAGTAAAATTGTATCG
CGCAAGCACAACAATACTGCTCTACTTATTCTCT
ATGGTGATGCGTG

Features
Complementary sticky end for binding Section 1
ATCGCTGAATTAT
Common sticky end to bind Next section
CGCAAGCACAACA
Enzyme tethering region and comp DNA sequence for enzyme attachment
CACGCATCACCAT
ATGGTGATGCGTG
Placement of Fluorescein labeled bases
T
Placement of alkyne labeled thymine
T

Section 3: 3 way junction

TGTTGTGCTTGCGCACGCATCACCATCGATACAATTTTATAGAGCAGTAT
ATAATTCAGCGATAGAGAATAAGTAAAATTGTATCG
ATAATTCAGCGATATACTGCTCTACTTATTCTCT
ATGGTGATGCGTG

Features

Complementary sticky end for binding Section 2
TGTTGTGCTTGCG
Common sticky end to bind Next section (*repeat of section 2*)
ATAATTCAGCGAT
Enzyme tethering region and comp DNA sequence for enzyme attachment
CACGCATCACCAT
ATGGTGATGCGTG
Placement of Fluorescein labeled bases
T
Placement of alkyne labeled thymine
T

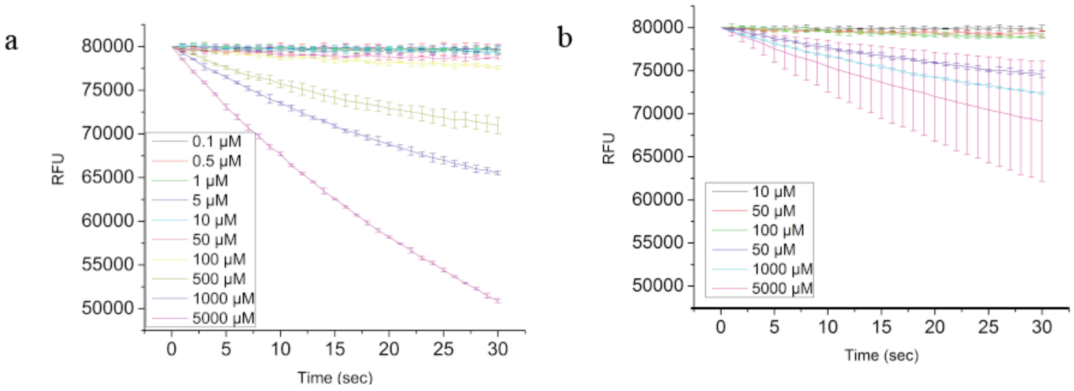

Figure S1. Comparison between nanosensors and butyrylcholinesterase in solution with fluorescein labeled DNA dendrimers. a) Kinetic traces of response of nanosensors to varying concentrations of acetylcholine. b) Kinetic traces of response of nanosensor fluorescent dendrimers with untethered butyrylcholinesterase in solution to varying concentrations of acetylcholine.

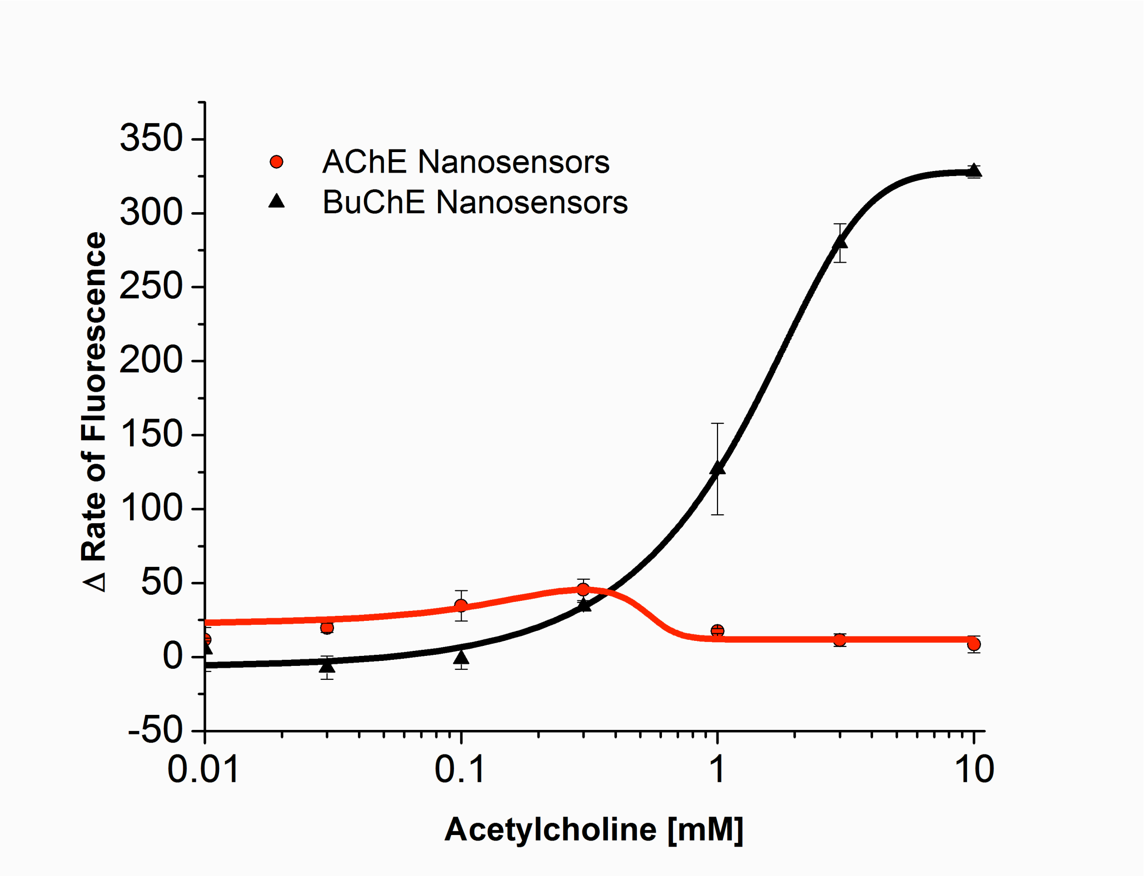

Figure S2. Responsiveness of acetylcholinesterase based nanosensors to acetylcholine. Rates (over the first 100 seconds) produced by acetylcholinesterase-based sensor exposed to acetylcholine in the low millimolar range were subject to substrate inhibition (*n*=3). Note, also, that the initial rates for acetylcholinesterase-based sensors are lower than the initial rates for the butyrylcholinesterase-based sensors.

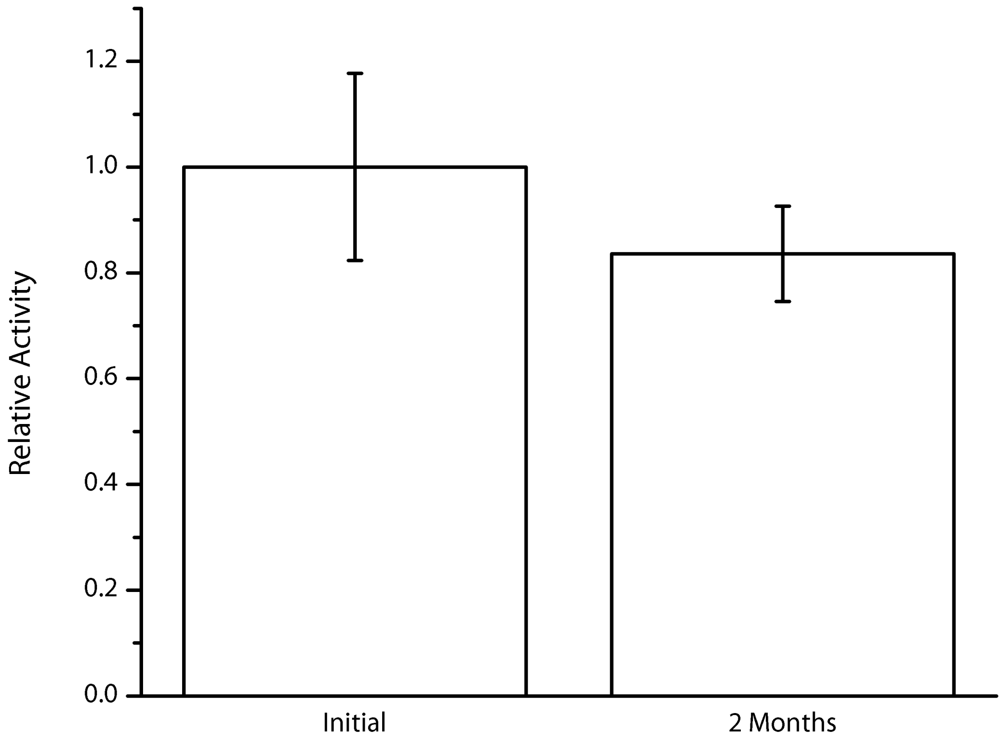

Figure S3. Nanosensor stability. Stability of the sensor was assessed by comparing the activity of the sensors towards 100 mM acetylcholine after 2 months of storage at 4°C. A comparison of the activity with the observed sensitivity of the sensors when they were freshly prepared suggested retention of 84% of the activity over the storage period.

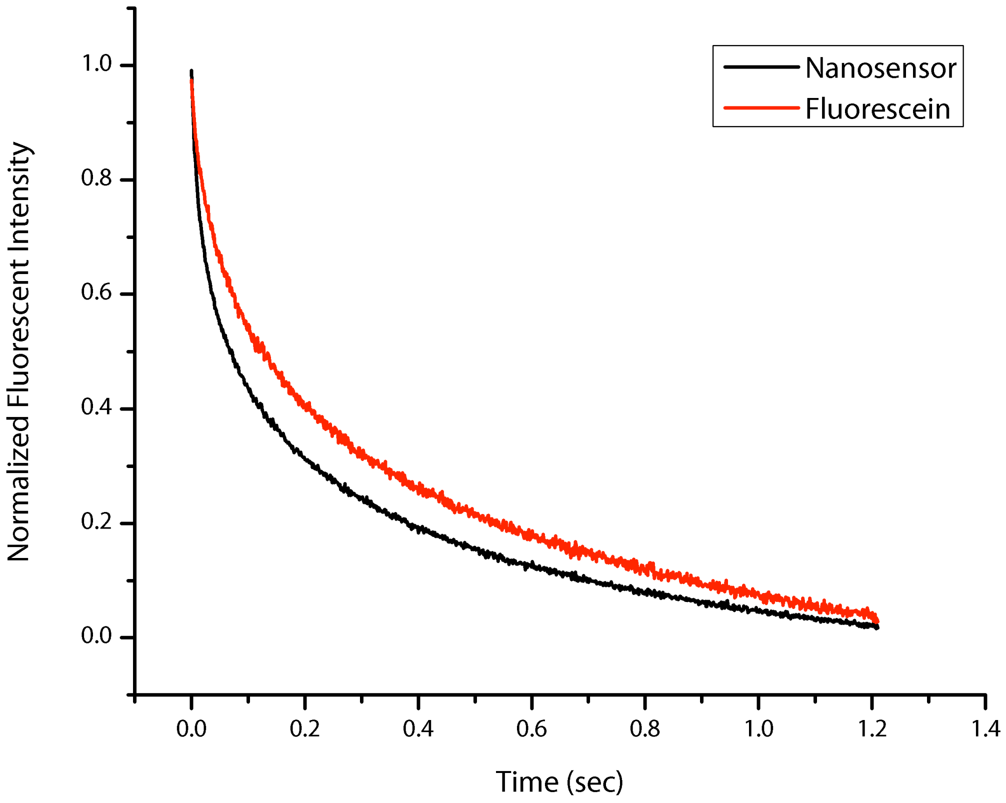

Figure S4. Nanosensor photobleaching. Stability of the fluorescein labeled sensor to photobleaching was assessed by exposing the nanosensors to a 488 nm laser. The photobleaching of the structure was found to be very comparable to fluorescein. The DNA dendrimers or fluorescein were embedded in a drop of 1.5% agar and excited with 488 nm, 50 mW laser light.

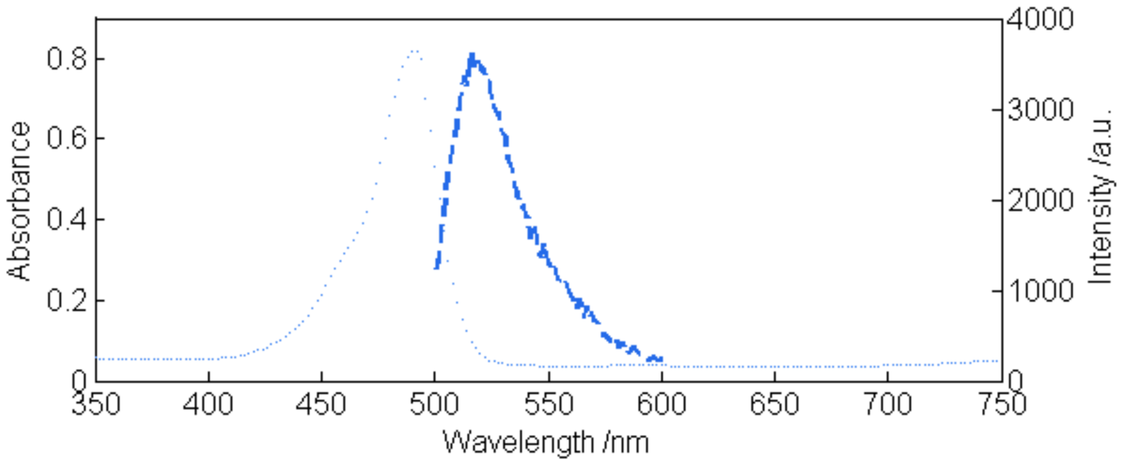

Figure S5. Quantum yield. Fluorescence quantum yield of the DNA dendrimer calculated as 0.71 with a|e - UV-Vis-IR Spectral Software 1.2, (FluorTools).

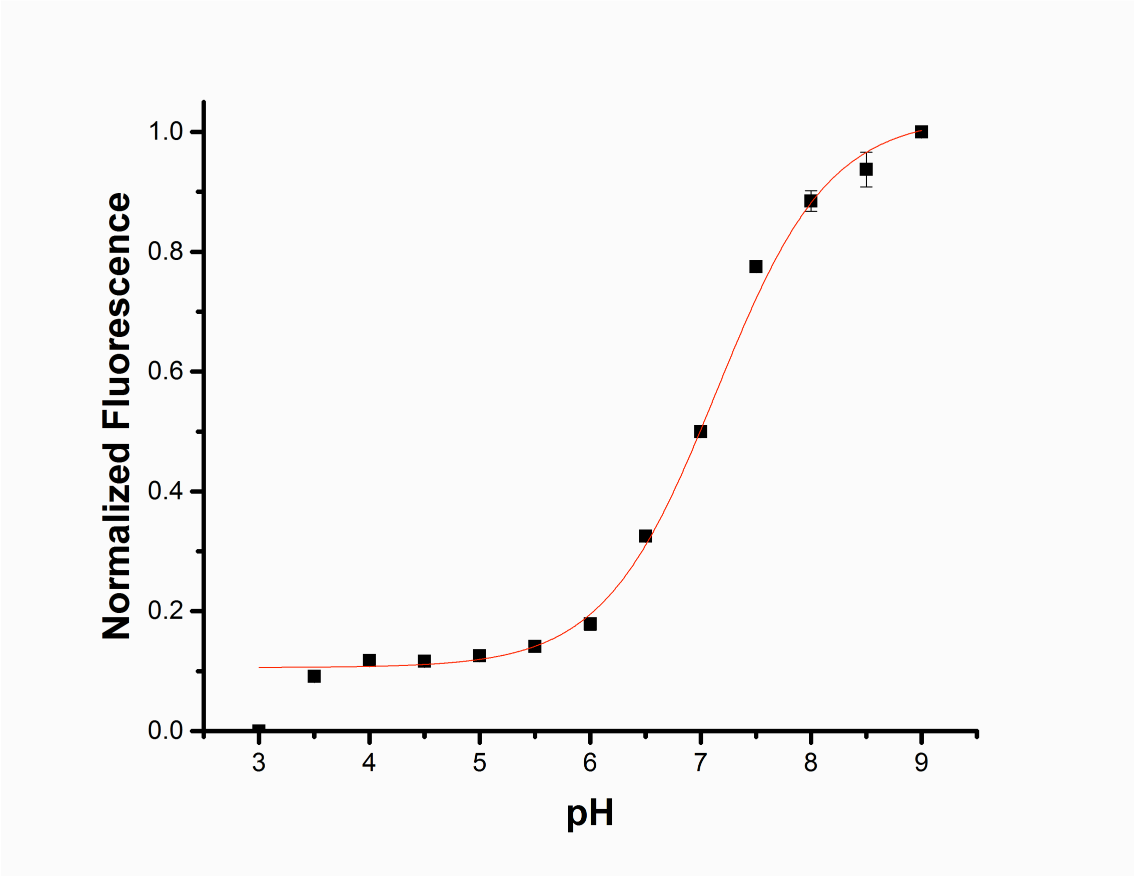

Figure S6. Dendrimer fluorescence in response to pH 3-9. DNA dendrimer fluorescent structures were tested against a pH range of 3-9, in triplicate. Using the fluorescein labeled DNA dendrimers the lower and upper limits of detection calculated as pH are 6.119 and 8.18, respectively.

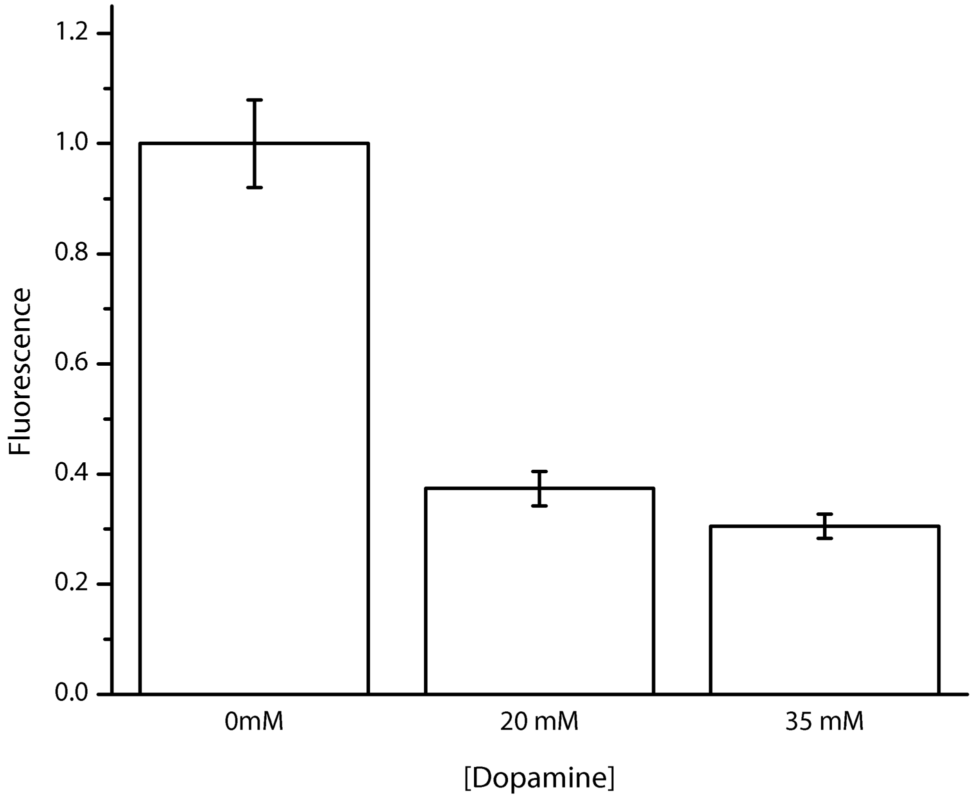

Figure S7. Interference with high concentrations of dopamine. Fluorescence interference by millimolar concentrations of dopamine in PBS and 1 mM ascorbic acid (*n*=3).

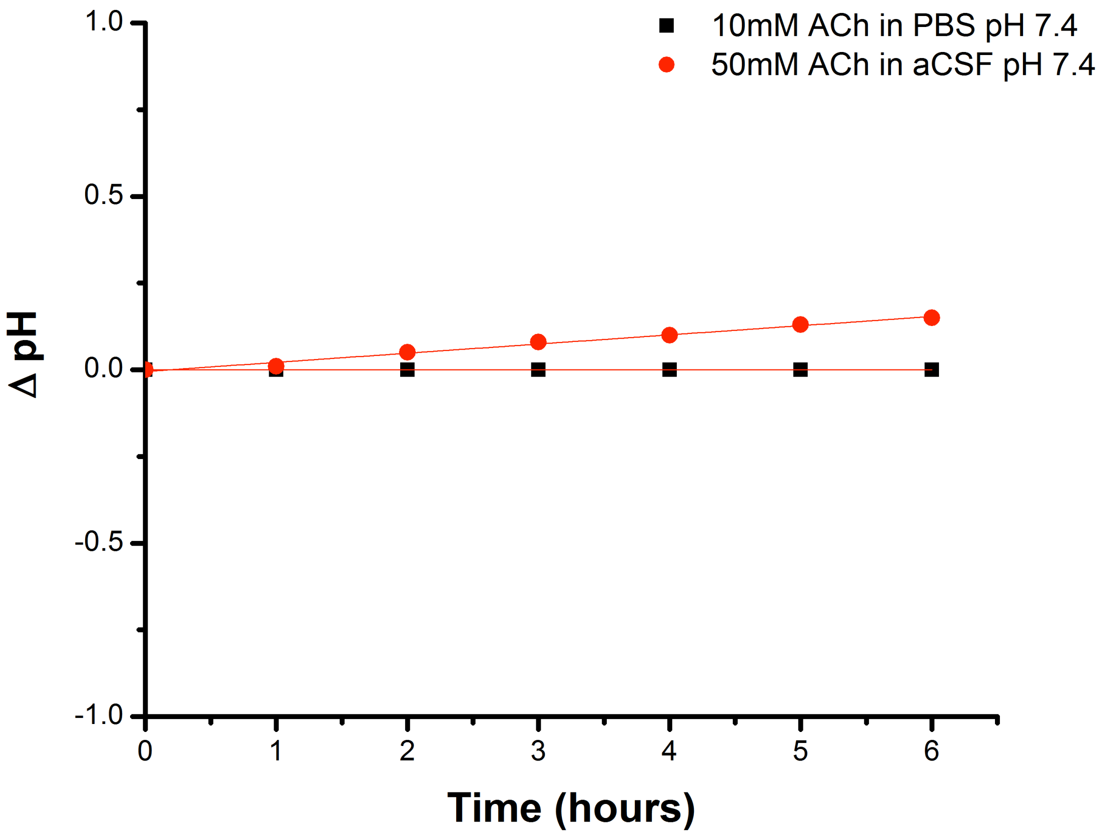

Figure S8. Changes in pH of acetylcholine (ACh) solutions over time. All calibration data associated with the sensors was taken in <4 hours using fresh acetylcholine solutions. (Cell and tissue data used 50mM acetylcholine in artificial cerebrospinal fluid (aCSF)). This shows the pH of acetylcholine solutions were constant at 10 mM concentrations and the pH slightly increased with 50 mM concentrations. Thus any changes seen due to pH increases would likewise increase the intensity of the sensor, where as the enzymatic response to acetylcholine decreases the fluorescence intensity. The correlation of fluorescence change per pHΔ0.1 is 3.9%; after 6 hours 50mM acetylcholine in aCSF has a 0.15 pH increase resulting in pH 5.85% increase in fluorescence.
